# Free oscillation rheometry monitoring of haemodilution and hypothermia and correction with fibrinogen and factor XIII concentrates

**DOI:** 10.1186/1757-7241-21-20

**Published:** 2013-03-22

**Authors:** Dag Winstedt, Nahreen Tynngård, Knut Olanders, Ulf Schött

**Affiliations:** 1 Consultant Anaesthetist, Lund University, Skane Universisty Hospital, Lund, 221 85 Lund, Sweden; 2 Division of Transfusion Medicine, Department of Clinical and Experimental Medicine, Faculty of Health Sciences, Linköping University, Linköping, Sweden; 3 Department of Clinical Immunology and Transfusion Medicine, County Council of Östergötland, Linköping, Sweden

**Keywords:** Free oscillation rheometry, Thrombelastography, Coagulation factor concentrate, Fibrinogen, Factor XIII, Haemodilution, Hypothermia, Coagulopathy, Hydroxyethyl starch, Ringer’s acetate solution

## Abstract

**Background:**

Haemodilution and hypothermia induce coagulopathy separately, but their combined effect on coagulation has not been widely studied. Fibrinogen concentrate can correct dilutional coagulopathy and has an additional effect when combined with factor XIII concentrate. However, their effect on dilutional coagulopathy concomitant with hypothermia has not been studied previously. Free oscillation rheometry – FOR (Reorox®) – is a novel viscoelastic haemostatic assay that has not been studied in this context before.

**Methods:**

Blood from 10 healthy volunteers was diluted by 33% with hydroxyethyl starch or Ringer’s acetate solutions. Effects of fibrinogen added *in vitro* with and without factor XIII were studied at 33°C and 37°C. Coagulation velocity (coagulation time) and clot strength (elasticity) were assessed with FOR. Coagulation was initiated *in vitro* with thromboplastin alone, or thromboplastin plus a platelet inhibitor.

**Results:**

Hydroxyethyl starch increased the coagulation time and decreased clot strength significantly more than Ringer’s acetate solution, both in the presence and absence of a platelet inhibitor. There was a significant interaction between haemodilution with hydroxyethyl starch and hypothermia, resulting in increased coagulation time. After addition of fibrinogen, coagulation time shortened and elasticity increased, with the exception of fibrinogen-dependent clot strength (i.e., elasticity in the presence of a platelet inhibitor) after hydroxyethyl starch haemodilution. Factor XIII had an additional effect with fibrinogen on fibrinogen-dependent clot strength in blood diluted with Ringer’s acetate solution. Hypothermia did not influence any of the coagulation factor effects.

**Conclusions:**

Both haemodilution and mild hypothermia impaired coagulation. Coagulopathy was more pronounced after haemodilution with hydroxyethyl starch than with Ringer’s acetate. Addition of fibrinogen with factor XIII was unable to reverse hydroxyethyl starch induced clot instability, but improved coagulation in blood diluted with Ringer’s acetate solution. Fibrinogen improved coagulation irrespective of hypothermia.

## Introduction

Exsanguination is the second most common cause of death in major trauma after central nervous system (CNS) injury [1,2]. To maintain an adequate circulatory blood volume and oxygen carrying capacity, traumatic and surgical haemorrhages are generally initially compensated for by administrating a combination of crystalloids, colloid solutions and packed red blood cells (PRBC). However, this results in haemodilution, hypothermia and acidosis, thus promoting the coagu-lopathy often seen in major haemorrhage [3,4]. In addition, synthetic colloid solutions such as hydro-xyethyl starch affect coagulation more than crystalloid solutions do [5,6].

Fibrinogen is the first coagulation factor to reach critical levels in major haemorrhage [7] and fibrinogen concentrate should be given in active bleeding when plasma levels reach 1.5 to 2.0 g/l [8]. This increases clot stability after haemodilution [9,10], reduces bleeding [11-13] and may improve survival [14,15]. The benefit of fibrinogen concentrate may be augmented by factor XIII (FXIII), since FXIII is responsible for cross-linking fibrin monomers [16].

Viscoelastic haemostatic assays (VHA), such as thrombe-lastography (TEG) and rotational thrombelastometry (ROTEM), may better guide blood component therapy in major bleeding compared to traditional coagulation tests [4,17]. According to analysis with VHAs, coagulation is impaired by both hypothermia [18,19] and haemodilution. In this study, we analysed coagulation with free oscillation rheometry (FOR), a novel viscoelastic haemostatic assay. The aim of our study was to investigate hypothermia- and haemodilution-induced coagulopathy measured with free oscillation rheometry, and to what extent this coagulopathy could be reversed with fibrinogen concentrate, combined with or without FXIII. Our hypotheses were that hypothermia and haemodilution would each impair coagulation measured with FOR and that the combination of hypothermia and haemodilution would interact, that is would impair coagulation synergistically. We also hypothesised that fibrinogen could reduce this coagulopathy, with an additional effect of FXIII.

## Materials and methods

### Study subjects and sampling

Ten healthy individuals without any known coagulation disorder (one woman and nine men, average age 43 years, with a mean haemoglobin level of 150 g/l) gave their written informed consent to donate 50 ml of blood. None of the subjects had taken any medication, including naturopathic preparations, during the seven days before blood collection. The Ethical Board, Lund, Sweden, approved the study (Registration Number: DNR 484).

Venipuncture was performed with a 21G needle and blood was collected into plastic Vacutainers®, mixing one part of citrate with nine parts of blood (BD, Plymouth, UK (citrate 0.129 mmol/l)). Blood was collected on two occasions on the same day per individual. The tubes were incubated for a minimum of 30 minutes and a maximum of four hours at a temperature of 33° or 37°C to ensure temperature equilibration.

### Haemodilution

Blood was diluted 33% with either Ringer’s acetate solution (RA) (Fresenius Kabi, Bad Homburg, Germany) or with 6% hydroxyethyl starch in saline (HES) (Mw 130 kDa, substitution 0.42, B. Braun, Melsungen, Germany). The solutions had a temperature of 33° or 37°C and the haemodiluted samples were kept at a temperature of 33° or 37°C until coagulation was analysed.

### Coagulation factor concentrates

Fibrinogen concentrate (Haemocomplettan®, now renamed Riastap®, CSL Behring, Marburg, Germany) was dissolved in distilled water (Fresenius Kabi) to a concentration of 20 g/l. FXIII concentrate (Fibrogammin®, CSL Behring) was dissolved in the solvent supplied by the manufacturer to a concentration of 62.5 U/ml. 120 μl of fibrinogen alone or 120 μl of fibrinogen supplemented with 15 μl of FXIII were added to a total sample volume of 3000 μl. These amounts correspond to therapeutic doses used in clinical practice: 4 g fibrinogen and 1550 U of FXIII to a 70-kg man, i.e., 55 mg fibrinogen and 22 U of FXIII per kg of body weight [20].

### Coagulation analysis

**Figure 1:**
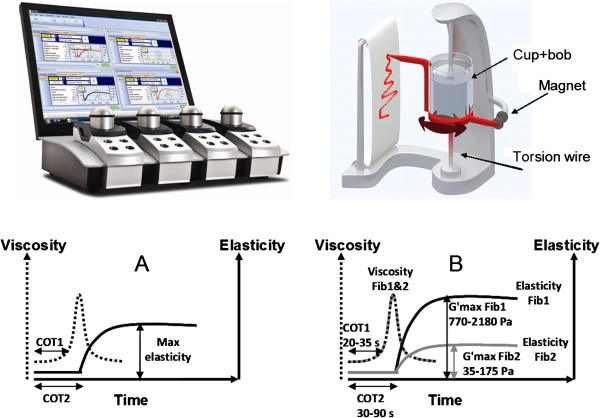
**Free oscillation rheometry.** The ReoRox G2 rheometer (upper left corner) and a schematic picture of its free oscillation sample cup (upper right corner). The magnet turns the sample cup around the torsion wire. Upon release, an oscillatory movement starts which is recorded by an optical detector. The change in damping generates a viscosity curve (dashed line) and the change in frequency generates an elasticity curve (full line) as shown in **A**. The damping (viscosity) of the oscillation increases as the sample coagulates until all is coagulated at which point the viscosity returns to baseline since the damping of the oscillation will not be affected anymore. This is followed by an increase in oscillation frequency (elasticity) when the platelets retract the clot. The height of the elasticity curve represents the strength of the clot. Variables detected are indicated with arrows (COT1- time to beginning of clot formation, COT2 – time to complete clot formation and Max elasticity (G'max) – maximum clot strength). **B** shows the differences in elasticity between FibScreen1 (full black line) and FibScreen2 (full grey line). The viscosity (COT1 and COT2) for FibScreen1 (dashed grey line) and FibScreen2 (dashed dotted line) is similar in a normal sample. Normal ranges are presented in the figure.

Clot formation and clot strength was studied with free oscillation rheometry (FOR), assessed with the ReoRox G2® rheometer (Medirox AB, Nyköping, Sweden). FOR, like thrombelastography, utilizes an oscillating movement to monitor coagulation. The sample is added to a reaction chamber, which consists of a gold-coated sample cup with a gold-coated cylinder (bob) suspended in the blood sample [21]. FOR uses a torsion wire system to set the sample into oscillation (Figure 1). A magnet pulls back the measuring head connected to the torsion wire. On release, the torsion wire will set the cup into free oscillation and its movement is recorded by an optical detector. The changes of damping and frequency of the oscillation correlates to viscosity and elasticity respectively, which are recorded as a viscosity curve and an elasticity curve (Figure 1).

The coagulation time (COT) is obtained from the viscosity curve. Coagulation time 1 (COT1) is the time to the start of clot formation when the initial strands of fibrin are formed [22]. Coagulation time 2 (COT2) is the time to complete clot formation after which elasticity starts building up and is equivalent to CT time for ROTEM. The clot strength in terms of maximum elasticity (G'max) is determined from the elasticity curve [23] and G'max is equivalent to MCF for ROTEM.

All analyses were performed according to the manufacturer’s instructions. Sample cups were placed into the heating blocks of the apparatus which were set to either 33° or 37°C. Temperatures were allowed to equilibrate and 1000 μl of blood sample was added to each sample cup. After re-calcification with 25 μl of 0.5 M CaCl_2_ (Medirox AB), coagulation was initiated with thromboplastin alone (FibScreen1, Medirox AB) or thromboplastin in the presence of abciximab (FibScreen2). Abciximab is a glycoprotein IIb/IIIa-receptor antibody and thus, inhibits platelet interaction with fibrinogen. Accordingly, FibScreen2 (Fib2) provided information about the functional fibrinogen concentration and fibrin stability of the clot. FOR tracings were analyzed for COT1, COT2 and G'max. G'max was determined for whole blood activated with FibScreen1 (Fib1) and for whole blood with inhibited platelets using Fib2 as activator. The difference in G'max between Fib1 and Fib2 was calculated as a measure of platelet-dependent clot strength. The normal ranges for the FOR parameters according to the manufacturer are presented in Figure 1. The coefficients of variation (CV) for the FOR parameters were 3% for COT1, 9% for COT2, 11% for Fib1 G'max and 15% for Fib2 G'max. The lowest detection level of G'max is set to 10 Pa in the software, which is well above the normal range for Fib2 G'max, and thus FOR should be able to detect low levels of functional fibrinogen concentrations.

### Statistical analysis

Kolmogorov-Smirnov tests were significant for several variables, showing that the data were not normally distributed. For this reason, all data were transformed, using the aligned rank transformation [24]. All transformed data were further analysed with repeated measures analysis of variance (ANOVA). Two separate ANOVAs were performed: a two-way ANOVA to study the influence of temperature and different solutions on coagulation and a three-way ANOVA to study the influence of added coagulation factors in hypothermia and haemodilution. Effects were presented as estimated marginal means, i.e., the mean of a factor averaged across all levels of the other factors. Where statistically significant differences were detected, further investigations of differences were made with contrast analysis. The Bonferroni correction was used to adjust for multiple comparisons. The aligned rank transformation was calculated with the free software ARTool. ANOVA statistical analysis was performed using PASW 18 (SPSS). Data were considered significant when the P-value was <0.05.

## Results

### FOR in hypothermia and haemodilution

In the first part of this study, the effects of mild hypothermia and haemodilution, alone or in combination, were investigated.

### Hypothermia

**Figure 2:**
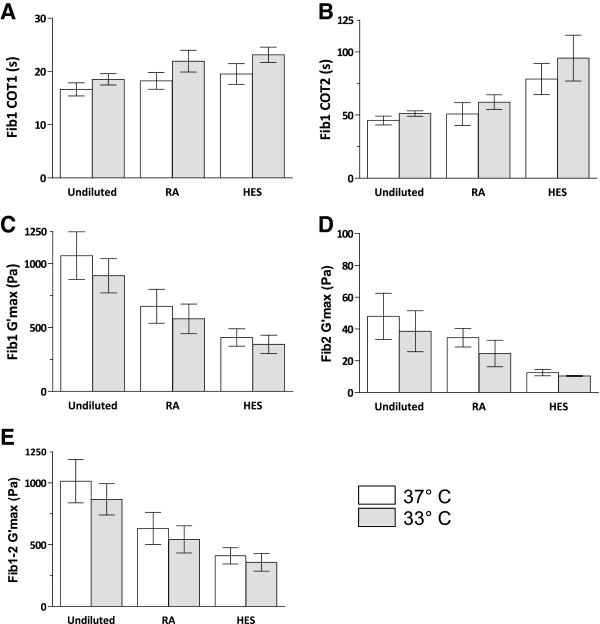
**Effects of haemodilution and hypothermia on coagulation.** Coagulation of undiluted, RA-diluted and HES-diluted blood at 33 and 37°C as assessed by FOR. The parameters COT1 (**A**), COT2 (**B**), maximum elasticity for FibScreen1 (**C**), maximum elasticity for FibScreen2 (**D**) and the difference in maximum elasticity for FibScreen1 and 2 (**E**) are shown. Coagulation time 1 (COT1) represents the beginning of clot formation and coagulation time 2 (COT2) a fully formed clot. G'max is the maximum elasticity of the fully formed clot. FibScreen1 (Fib1) denotes that thromboplastin was added to the sample and FibScreen2 (Fib2) indicates that thromboplastin and abciximab were added, the latter a potent platelet inhibitor. Thus, Fib1 G'max represents whole blood clot strength and Fib2 G'max fibrinogen-dependent clot strength. FS1 – FS2 G'max was calculated to represent platelet-dependent clot strength. N = 10. Bars are 95% CI.

Mild hypothermia impaired all the measured aspects of coagulation (Table 1 and Figure 2). Both the time to the initiation phase (COT1) of coagulation and the time to complete clot formation (COT2) were significantly prolonged in hypothermia. Also, the final clot strength weakened with hypothermia, measured as a decrease in G'max. G'max decreased in whole blood (Fib1), in whole blood with inhibited platelets (Fib2), as well as the calculated platelet-dependent (the difference between Fib1 and Fib2) G'max.

**Table 1:** Effects of hypothermia and haemodilution on coagulation measured with free oscillation rheometry (FOR)

	**Temperature**			**Solution**					
**Variable**	**37°C**	**33°C**		**Undiluted**	**RA**		**HES**		
COT1 (s)	18.1	21.2	**	17.6	20.1	**	21.3	***	
COT2 (s)	58.3	68.8	***	48.5	55.5	**	86.7	**	†
G'max Fib1 (Pa)	716	613	***	983	617	***	395	***	†††
G'max Fib2 (Pa)	31.7	24.5	***	43.3	29.5	***	11.5	***	†††
G'max Fib1-2 (Pa)	685	589	**	940	587	***	383	***	†††

Effects on coagulation of hypothermia (33°C versus 37°C) and 33% haemodilution with RA or HES compared to undiluted blood, presented as estimated marginal means. COT1 and COT2 are coagulation time 1 and 2, respectively. The maximal clot strength is presented as G'max for Fib1 (without platelet inhibition) and G'max for Fib2 (with platelet inhibition). Platelet-dependent clot strength is presented by the difference between G'max for Fib1 and that for Fib2, denoted as G'max Fib1-2.

** and *** stands for p < 0.01 and p < 0.001, respectively, when the two temperatures were compared or when diluted blood (RA or HES) was compared to undiluted blood. †† and ††† stands for p < 0.01 and p < 0.001, respectively, when haemodilution with RA was compared to HES. See text for details on interactions. N = 10.

### Haemodilution

Haemodilution with RA or HES impaired all the measured aspects of coagulation (Table 1 and Figure 2). Coagulation times (COT1 and COT2) were prolonged and G'max decreased in whole blood (Fib1), in whole blood with inhibited platelets (Fib2), as well as the calculated platelet-dependent clot strength. Haemodilution with HES consistently impaired coagulation more than haemodilution with RA. However, this difference was not statistically significant for COT1 (Table 1).

### Interaction of hypothermia and haemodilution

A significant interaction effect (synergy) was shown for COT2 between hypothermia and haemodilution with HES compared to undiluted blood (p = 0.035; hypothermia X haemodilution (undiluted vs. HES)). COT2 was prolonged after haemodilution with HES and even more when HES haemodilution was combined with hypothermia (Figure 2). Thus, hypothermia and haemodilution with HES prolonged COT2 more than explained by their respective additive effects. Interestingly, this synergy could not be found between hypothermia and haemodilution with RA.

Significant interaction effects were also shown for Fib2 G'max between hypothermia and haemodilution with HES compared to either undiluted blood (p < 0.001) or blood diluted with RA (p = 0.003; Table 2). However, these statistically significant interactions were not proofs of synergy. Instead they showed, as seen in Figure 2, that hypothermia had less additional effect on Fib2 G'max in blood diluted with HES, as compared to undiluted blood or blood diluted with RA. The reason for this is probably technical, since the FOR instrument never yields G'max values of <10 Pa. After haemodilution with HES, FOR is simply unable to detect any additional decreases in Fib2 G'max.

**Table 2:** Interaction effects of (a) temperature and the type of solution in haemodilution and (b) the type of solution in haemodilution and added coagulation factor

	**(a) Temperature X solution**			**(b) Solution X coagulation factor**		
	**Und**	**Und.**	**RA**	**Control vs.**	**Control vs.**	**Fibrinogen vs.**
**Variable**	**vs. RA**	**vs. HES**	**vs. HES**	**Fibr.**	**Fibr. + FXIII**	**Fibr. + FXIII**
**COT1 (s)**	**-**	**-**	**-**	**-**	**-**	**-**
**COT2 (s)**	**-**	**0.035**	**-**	**0.001**	**0.002**	**-**
**G'max Fib1 (Pa)**	**-**	**-**	**-**	**-**	**-**	**-**
**G'max Fib2 (Pa)**	**-**	**<0.001**	**0.003**	**<0.001**	**<0.001**	**<0.001**
**G'max Fib1-2 (Pa)**	**-**	**-**	**-**	**-**	**-**	**-**

Significances (p-values) of the interaction effects. The left half of the table (a) shows the interaction between temperature (33°C versus 37°C) and solution (Und. = undiluted, RA, or HES), according to a two-way ANOVA (Temperature X Solution).

The right half of the table (b) shows the interaction between solution (RA or HES) and added coagulation factor (Control, i.e., no addition of coagulation factor, fibrinogen or fibrinogen with FXIII), according to a three-way ANOVA (Temperature X Solution X Coagulation Factor). Not shown from the latter ANOVA are the interactions of Temperature X Coagulation Factor. None of these interactions were significant. The maximal clot strength is presented as G'max for Fib1 (without platelet inhibition) and G'max for Fib2 (with platelet inhibition). Platelet-dependent clot strength is presented by the difference between G'max for Fib1 and that for Fib2, denoted as G'max Fib1-2. RA is Ringer’s acetate solution. HES is hydroxyethyl starch in saline. Interactions marked with - were not significant. N = 10.

### FOR in hypothermia and haemodilution with coagulation factor substitution

In the second part of this study, the effects on coagulation of adding factor concentrate (fibrinogen with or without FXIII) in hypothermia and haemodilution were studied.

### Coagulation factor substitution

**Figure 3:**
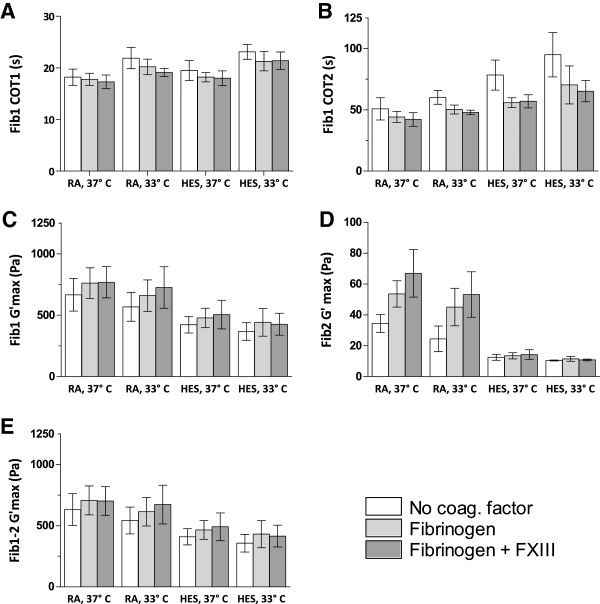
**Effects of factor concentrate addition on coagulation.** Coagulation of RA-diluted and HES-diluted blood at 33 and 37°C upon addition of factor concentrates (fibrinogen or fibrinogen + FXIII) as assessed by FOR. The parameters COT1 (**A**), COT2 (**B**), maximum elasticity for FibScreen1 (**C**), maximum elasticity for FibScreen2 (**D**) and the difference in maximum elasticity for FibScreen1 and 2 (**E**) are shown. Coagulation time 1 (COT1) represents the beginning of clot formation and coagulation time 2 (COT2) a fully formed clot. G'max is the maximum elasticity of the fully formed clot. FibScreen1 (Fib1) denotes that thromboplastin was added to the sample and FibScreen2 (Fib2) indicates that thromboplastin and abciximab were added, the latter a potent platelet inhibitor. Thus, Fib1 G'max represents whole blood clot strength and Fib2 G'max fibrinogen-dependent clot strength. FS1 – FS2 G'max was calculated to represent platelet-dependent clot strength. N = 10. Bars are 95% CI.

The overall effect of substitution with coagulation factors is presented in Table 3 and Figure 3. The addition of fibrinogen significantly improved all FOR parameters, except the initiation of coagulation (COT1). However, a small additional effect when combining fibrinogen with FXIII shortened COT1 significantly compared to the control group without coagulation factors added. Apart from this effect of FXIII on COT1, fibrinogen-dependent clot strength, i.e., Fib2 G'max was the only parameter to benefit further by combining fibrinogen with FXIII. Thus, Fib2 G'max increased significantly after adding fibrinogen and rose significantly more when fibrinogen was combined with FXIII. However, as seen in Figure 3, Fib2 G'max increased with the addition of fibrinogen (with or without FXIII) only in RA-diluted blood (see below).

**Table 3:** Effects of added coagulation factors on coagulation measured with free oscillation rheometry (FOR)

	**Coagulation Factor**					
**Variable**	**Control**	**Fibrinogen**		**Fibrinogen + FXIII**		
COT1 (s)	20.7	19.4		19.0	**	
COT2 (s)	71.1	55.2	***	53.1	***	
G'max Fib1 (Pa)	506	585	**	606	**	
G'max Fib2 (Pa)	20.5	31.0	***	36.3	***	††
G'max Fib1-2 (Pa)	485	554	**	570	**	

Effects on coagulation of the coagulation factors fibrinogen, fibrinogen with factor XIII (Fibrinogen + FXIII) or no addition of coagulation factor (Control). Data are estimated marginal means. COT1 and COT2 are coagulation time 1 and 2, respectively. The maximal clot strength is presented as G'max for Fib1 (without platelet inhibition) and G'max for Fib2 (with platelet inhibition). Platelet-dependent clot strength is presented by the difference between G'max for Fib1 and that of Fib2, denoted as G'max Fib1-2. ** and *** stands for p < 0.01 and p < 0.001, respectively, when the addition of coagulation factor was compared to the control group. †† and ††† stands for p < 0.01 and p < 0.001, respectively, when the addition of fibrinogen alone was compared to the addition of fibrinogen with FXIII. See text for details on interactions. N = 10.

### Interaction of type of solution in haemodilution and addition of coagulation factor

There were significant interactions between the type of solution in haemodilution and added coagulation factor for COT2 and Fib2 G'max.

Adding fibrinogen (p = 0.001) or fibrinogen with FXIII (p = 0.002) decreased COT2 more in blood diluted with HES than with RA. There was no significant difference between fibrinogen and fibrinogen with FXIII.

On the contrary, Fib2 G'max increased more after adding fibrinogen (p < 0.001) or fibrinogen with FXIII (p < 0.001) in blood diluted with RA than in that with HES. In fact, Fib2 G'max did not improve at all in blood diluted with HES (Figure 3). In RA diluted blood, Fib2 G'max increased most when fibrinogen was combined with FXIII (p < 0.001).

### Interaction of hypothermia and addition of coagulation factor

There were no significant interactions between hypo-thermia and the addition of any combination of coagulation factors.

## Discussion

This study shows that cooling blood to 33°C and haemodilution with HES interacts to impair clot formation. We also found that fibrinogen substitution was effective also at 33°C, alone or in combination with haemodilution. The type of solution in haemodilution, however, did affect the reversal effect of fibrinogen in dilutional coagulopathy. After adding fibrinogen, clot propagation (COT2) improved more in HES haemodilution, while fibrinogen-dependent clot strength (Fib2 G'max) increased in RA haemodilution only, and not at all in HES haemodilution. Furthermore, combining fibrinogen with FXIII had an additional effect on fibrinogen-dependent clot strength (Fib2 G'max) with RA haemodilution only. Apart from these principle findings, we also confirmed the results of previous studies with other viscoelastic haemostatic assays that showed that both hypothermia and haemodilution independently impaired coagulation, and that HES impaired coagulation more than RA.

VHAs such as thrombelastography, measure coagulation in whole blood rather than in plasmaand they are therefore able to detect interactions between platelets, red blood cells and coagulation factors. Thus, VHAs more accurately describe coagulation in vivo and they are better predictors of bleeding than traditional measures of coagulation [25]. FOR’s ability to measure viscosity changes enables detection of the start of clot formation (COT1), which is in contrast to with thrombelastography. FOR uses free oscillation, which results in less strain on the clot as compared with thrombelastography. Compared to maximal clot strength measured with thrombelastography, the corresponding measure with FOR (Fib1 G'max) is more dependent on platelets than on factors affecting the fibrinogen polymerization, i.e. fibrinogen concentration and FXIII [21]. Thus, FOR may provide new information on the effect of hemodilution and hypothermia on coagulation.

In concordance with earlier studies with VHAs, we found that hypothermia impaired clot formation variables [18,26]. In addition, we observed that clot strength was decreased even by mild hypothermia, which other studies failed to detect [19,27]. Previous studies only noted this decrease in clot strength during moderate to severe hypothermia [18,26]. The different measuring principles of ROTEM and FOR might be the cause of the different effects observed on clot strength by hypothermia. The possible higher sensitivity of clot strength variables measured with the FOR than with other methods is interesting. However, the decrease in clot strength observed from 37° to 33°C is moderate and unlikely to have any clinical implications.

Our results demonstrated that haemodilution with either HES or RA compromised coagulation, and that the effect of HES was more pronounced. This is in accordance with previous studies where HES was shown to affect coagulation more than RA both *in vitro*[28,29] and *in vivo*[30,31]. It is believed that HES, in contrast to crystalloids, also impair fibrinogen/fibrin polymerization [29].

Not only platelets are important cellular components of global haemostasis, but also erythrocytes play an important role [32]. The role of the haematocrit level has been previously studied. In vitro studies with FOR and TEG, where the platelet counts were held constant, showed increased clot strengths with decreasing haematocrit levels [21,33]. This increase in clot strength may be due to increasing fibrinogen levels after haemodilution with plasma. Ogawa recently also showed an increase in clot strength after haemodilution with plasma [34]. However, at a given fibrinogen concentration, clot strength instead improved with falling haematocrit levels. Thus, fibrinogen levels seem to be far more important than haematocrit level for the final clot strength.

Other studies have not found any interaction effects between hypothermia and haemodilution, as assessed by coagulation time and clot strength [35,36]. We discerned no interactions for the clot strength variables, but we did find significant interaction effects between hypothermia and haemodilution with HES in the time to complete clot formation (COT2). There was also a tendency towards interaction between hypothermia and haemodilution with RA for the time to complete clot formation (COT2), as well as between hypothermia and haemodilution with both solutions for the initiation of clot formation (COT1). FOR and thrombelastography are not completely comparable methods. For example, in contrast to thrombelastography, FOR can measure viscosity changes and discerns the coagulation time from the changes in viscosity. This may explain the inability of thrombelastography to detect any interaction effects on variables describing the early phases of coagulation**.**

Substitution with fibrinogen concentrate in dilutional coagulopathy has been demonstrated to effectively increase clot strength [9,10,13] and to reduce bleeding [11,37]. A high ratio of fibrinogen to red blood cell transfusion is associated with improved survival rates [38]. In this study, we found that fibrinogen increased clot strength and shortened the coagulation time (COT1 and COT2). The reduction in COT1, though, was very small and significant only when fibrinogen was combined with FXIII. These results are in agreement with the aforementioned studies with ROTEM, where addition of fibrinogen increased clot strength, while the coagulation time (CT) failed to improve after addition of fibrinogen [9,13]. Interestingly, fibrinogen supplementation decreased COT2 significantly more in haemodilution with HES than in that with RA, while fibrinogen-dependent clot strength increased more in haemodilution with RA. In fact, Fib2 G'max did not increase at all after adding fibrinogen or fibrinogen combined with FXIII in haemodilution with HES. In other words, fibrinogen substitution reversed the early phases of the coagulation regardless of the presence of starch, while haemodilution with HES made fibrinogen unable to improve clot strength. One may speculate that higher doses of fibrinogen may be required to improve also clot strength after HES haemodilution.

FXIII’s activity is diminished by haemodilution [39]. FXIII is ineffective at correcting dilutional coagulopathy *in vitro*[10], but has been shown to decrease post-operative chest-drain bleeding after cardiac surgery [40]. Combining fibrinogen with FXIII in RA haemodilution had an additional on Fib2 G'max but no effect on Fib1 G'max, as discussed above. The additional increase of Fib1 G'max after adding FXIII together with fibrinogen is small (not significant) and will be masked by the platelets when assessing Fib1 G'max. Therefore, analysis of Fib2 G'max will add important information on factors affecting the fibrinogen polymerization.

The findings of this study emphasise the importance of reversing hypothermia and avoiding haemodilution. As previously concluded, it is reasonable to avoid HES in cases of major haemorrhages. Our study showed that the addition of fibrinogen was also beneficial in mild hypothermia and thus, patients are not required to be warmed before infusing fibrinogen. Moreover, if HES has been used in seriously bleeding patients, higher doses than those given in this study are probably needed to re-establish adequate clot strength. Finally, combining fibrinogen with FXIII may be worthwhile in RA induced coagulopathy, since this combination improved fibrinogen-dependent clot strength (Fib2 G'max) more than fibrinogen alone in this in vitro study. However, controlled clinical studies need to be undertaken with FOR, to establish correct doses of fibrinogen and factor XIII in dilutional coagulopathy.

In contrast to other studies, we studied the combination of concurrent hypothermia, haemodilution and coagulation factors. We chose to study *mild* hypothermia and *moderate* haemodilution, since we wished to study a common and clinically relevant situation. Unfortunately, this decreased the power to detect any statistical differences. Furthermore, FOR is not a widely used method in investigations of coagulopathy in bleeding patients. However, comparison with studies using thrombelastography (ROTEM and TEG) is logical, since FOR is very similar to thrombelastography and these methods are good predictors of perioperative bleeding. There are several other limitations to performing in vitro haemodilution studies using citrated blood, as is the case in this study. The situation in vitro may not accurately represent the situation in vivo. For example, in vitro models do not account for shear stress, release of tissue factor by the endothelium, and activation of procoagulation or fibrinolytic pathways in response to tissue trauma. Our study was performed in blood from healthy volunteers, whereas trauma patients may exhibit a wide range of coagulopathies.

## Conclusions

In conclusion, this in vitro study shows that hypothermia at 33°C combined with 33% haemodilution, especially with HES, interact to impair coagulation variables measured with free oscillation rheometry (FOR). These effects were reduced by adding fibrinogen regardless of hypothermia at 33°C. After haemodilution with RA, coagulation variables were improved by fibrinogen, but clot strength was not improved with fibrinogen after haemodilution with HES. Further, FXIII had an additional effect on the effects of fibrinogen substitution, at least on the fibrinogen-dependant clot strength. In summary, higher fibrinogen doses than previously recommended may be required to reverse HES-induced coagulopathy and the combination of fibrinogen with factor XIII may be beneficial. Further clinical investigations are needed to establish the optimal dose of fibrinogen and to elucidate the role of FXIII in dilutional coagulopathy.

## Abbreviations

COT: Clotting time; FXIII: Coagulation factor XIII; Fib1/Fib2: Fibscreen1/Fibscreen 2 (tests with FOR); FOR: Free oscillation rheometry; G’max: Maximum elasticity; HES: Hydroxyethyl starch; RA: Ringer’s acetate solution; ReoRox G2®: FOR apparatus used in this study; ROTEM®: Rotational thrombelastometry; TEG®: Thrombelastography (both trademark and generic name of technology); VHA: Viscoelastic haemostatic assay e.g., ROTEM, TEG and FOR.

## Competing interests

US has received grants from CSL Beehring. At the time of the study, NT was part time employed by Medirox AB, Nyköping, Sweden. DW and KO have no competing interests.

## Authors’ contributions

DW carried out the laboratory work, performed the statistical analysis and drafted the manuscript including all tables and figures. NT drafted the manuscript and participated in the design of tables and figures. KO drafted the manuscript and participated in the design of tables. US designed the study and drafted the manuscript, and is the senior author of this study. All authors read and approved the final manuscript.
